# When to keep it simple – adaptive designs are not always useful

**DOI:** 10.1186/s12916-019-1391-9

**Published:** 2019-08-02

**Authors:** James M. S. Wason, Peter Brocklehurst, Christina Yap

**Affiliations:** 1 Institute of Health and Society, Newcastle University, Baddiley-Clark Building, Richardson Road, Newcastle upon Tyne, NE2 4AX UK; 2 MRC Biostatistics Unit, University of Cambridge, Cambridge, UK; 3 Birmingham Clinical Trials Unit, University of Birmingham, Birmingham, UK; 4 Cancer Research UK Clinical Trials Unit, University of Birmingham, Birmingham, UK; 5 Clinical Trials and Statistics Unit, The Institute of Cancer Research, London, UK

**Keywords:** Adaptive design, Clinical trials, Efficiency, Patient benefit

## Abstract

**Background:**

Adaptive designs are a wide class of methods focused on improving the power, efficiency and participant benefit of clinical trials. They do this through allowing information gathered during the trial to be used to make changes in a statistically robust manner – the changes could include which treatment arms patients are enrolled to (e.g. dropping non-promising treatment arms), the allocation ratios, the target sample size or the enrolment criteria of the trial. Generally, we are enthusiastic about adaptive designs and advocate their use in many clinical situations. However, they are not always advantageous. In some situations, they provide little efficiency advantage or are even detrimental to the quality of information provided by the trial. In our experience, factors that reduce the efficiency of adaptive designs are routinely downplayed or ignored in methodological papers, which may lead researchers into believing they are more beneficial than they actually are.

**Main text:**

In this paper, we discuss situations where adaptive designs may not be as useful, including situations when the outcomes take a long time to observe, when dropping arms early may cause issues and when increased practical complexity eliminates theoretical efficiency gains.

**Conclusion:**

Adaptive designs often provide notable efficiency benefits. However, it is important for investigators to be aware that they do not always provide an advantage. There should always be careful consideration of the potential benefits and disadvantages of an adaptive design.

## Background

There is a great desire to improve the efficiency of clinical trials, which are expensive, time-consuming and contribute to the high cost of drug development [1]. One initiative to address this is the use of adaptive designs, which provide the opportunity to use data accrued during the trial to make relevant changes [2, 3]. Potential changes relate to the allocation of patients to specific treatment arms, enrolment criteria and the target sample size. Generally, the scope of potential changes is clearly laid out prior to trial commencement.

Adaptive designs can lead to improved efficiency (either fewer participants on average to achieve the same level of statistical power to detect a true treatment effect, or higher power for the same number of participants) and trial attractiveness to enrolled participants (e.g. by closing ineffective arms earlier and allocating more participants to treatments that have shown more promise from data accrued so far). Some argue that adaptive trials are more ethical, although this is a complex and controversial statement. We refer the reader to work on this subject (e.g. [4, 5]) and restrict our attention to the narrower context of what number and proportion of participants receive ineffective treatments.

**Fig. 1:**
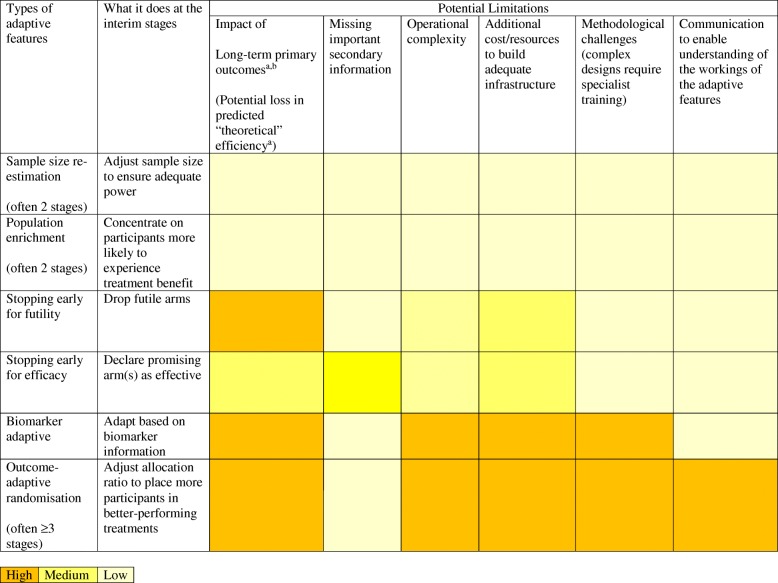
Assessment of the impact of potential adaptive design limitations on different types of adaptive trial features. ^a^Assuming no pause in recruitment; ^b^Primary endpoint with an intermediate observation period. Some adaptive designs were extracted from Table 1 from Pallmann et al. [2] (with the removal of dose-finding designs); MAMS have several arms and could include any of the above adaptive features at the interim stages with added complexity

Adaptive designs have been used in a range of settings, including a small trial testing new imaging techniques [6], a two-arm trial investigating cannabis use in multiple sclerosis [7] and ongoing large oncology trials testing a pipeline of treatments [8, 9]. A range of adaptations are possible, as shown in Fig. 1 and elaborated on by Pallmann et al. [2]. Several reviews have been published investigating properties of published adaptive designs, including study characteristics [10, 11], and views from stakeholders on their utility [12].

As researchers with an interest in methodology of adaptive designs, we are often approached to collaborate to apply them in practice. It has been hugely pleasing to us to see increasing enthusiasm amongst clinical investigators for their use. This is often for good reasons, with the benefits of adaptive designs being compelling. However, in some situations, the drawbacks of using an adaptive approach outweigh the benefits. Although reasons for this have been mentioned in disparate papers, primarily statistical, we believe this issue is not sufficiently emphasised in the literature that promotes adaptive trials. Here, we provide some guidance on situations where we believe the trial design should be kept more straightforward.

## Long-term outcomes

For adaptive designs to improve the efficiency of a trial, the information at an interim analysis must be useful for predicting what would occur if the trial were to continue to the end. As an example, consider a multi-arm, multi-stage (MAMS) trial that allows several experimental treatments to be tested against a shared control, and for experimental treatment arms to be dropped from the trial early if one or more treatments are not showing promising evidence of benefit. This is potentially advantageous to future participants in the trial who are less likely to be allocated to an ineffective treatment and also allows increased efficiency. Both of these advantages are strongly dependent on making a reliable decision at the interim analysis and to avoid wrongly dropping an actually effective treatment. In order to do this, the decision needs to be made on outcome information from a reasonable number of participants. This requires that the outcome being used to decide whether to drop one or more treatment arms should be observed sufficiently quickly (compared to the planned recruitment length of the trial). If it is not, the trial may be complete before a sufficient number of outcomes have been observed to make any material difference to the trial. If an intermediate outcome is being used, this must (1) be highly predictive of the primary outcome, accepting that the association between the intermediate outcome and the substantive outcome may be altered by the intervention [13], and (2) it must also be observed quickly enough to allow modification of the trial design. If neither of these is the case, then one of two possibilities will occur, namely (1) at the interim analysis, there will be many participants who have been recruited but are not yet in a position to contribute information at the interim analysis; or (2) recruitment will be paused at the interim analysis until all recruited participants have been assessed. Neither of these possibilities is desirable. The first will mean that participants who are recruited but not yet assessed cannot contribute to, or benefit from, the interim analysis; the second will mean that the trial will take much longer, considering even that it is feasible to pause recruitment (it usually is not).

Methodological papers often do not consider the rate of enrolment versus the length of follow-up for outcomes when quantifying the efficiency advantages of adaptive designs. Often, participant outcomes are assumed to be available immediately after recruitment – clearly unrealistic in many situations – with the implication that the reported benefits of these designs in the literature are overinflated. The various adaptive study designs are likely to be differentially affected by this delay (Fig. 1). Two-arm sample size re-assessment designs, which allow the planned sample size to be increased after an interim analysis, are less affected, whereas MAMS or outcome-adaptive randomisation designs (more participants are randomised to better performing arms) are more affected [14, 15].

**Fig. 2:**
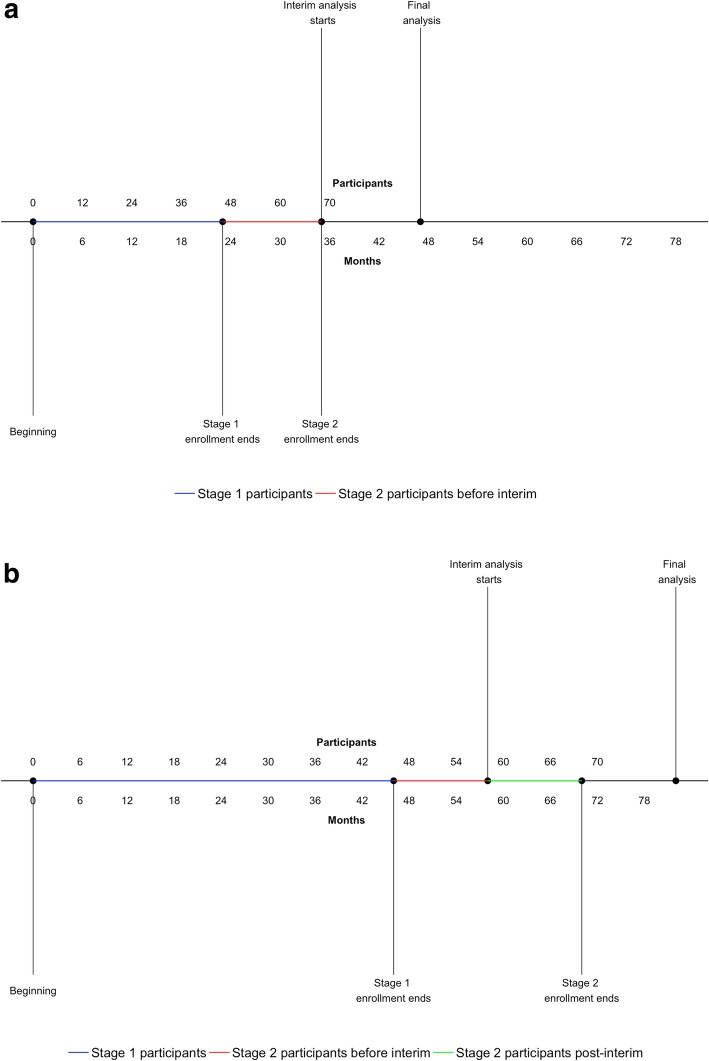
Timeline of the first example trial (ISRCTN 11889464) if participants were recruited at a rate of two per month (**a**) and one per month (**b**). Stage 1 enrolment represents the pre-planned number of individuals who would provide information at the interim analysis. The red part of the x axis denotes stage 2 participants who are recruited prior to the interim analysis being started; the green part denotes stage 2 participants who are recruited after the interim analysis starts (and who thus may benefit from the adaptive design)

We consider two examples to illustrate this point. The first is Immunotace, an ongoing randomised phase II trial in Birmingham Cancer Research UK Clinical Trials Unit assessing the benefit of the addition of dendritic cells in an immunotherapy trial in hepatocellular carcinoma (ISRCTN: 11889464). Originally, this trial had been planned with an adaptive design. The primary outcome of progression-free survival rate at 12 months was to be used to allow the potential for stopping at the interim analysis for futility after 23 participants per arm. With the projected recruitment rate (two participants/month) the trial would have reached its full sample size of 70 patients by the time the first stage participants reached 12 months follow-up of progression-free survival. The timeline of the trial if participants were recruited as planned (Fig. 2a, two participants per month) and at a slower rate (Fig. 2b, one participant per month) clearly shows that, in the first case, the pre-planned adaptation would have been pointless, with no possibility to stop participants being exposed to a potentially ineffective therapy. Instead, this trial was re-designed using a simpler, single-stage design and efficiency was gained by changing the primary outcome to a time-to-event outcome. The adaptation would have provided more utility if either the recruitment rate had been slower (e.g. one patient per month; Fig. 2b) or there had been an informative intermediate endpoint observed more quickly.

The second example is the TAILoR trial [16], which is a four-arm, two-stage trial testing three doses of telmisartan for the reduction of insulin resistance in HIV-positive individuals. The arms are treated as distinct (as opposed to applying a dose–response model) as there was reason to believe that the relationship between dose and outcome would be complex. The outcome was change in ﻿Homeostatic Model Assessment – Insulin Resistance from baseline to 24 weeks. The total planned recruitment length, according to the ISRCTN registration (ISRTCTN51069819), was 28 months. The design used the methodology of Magirr et al. [17] to control the total chance of making any type I error at 5%. The target sample size was set to be 336 patients with a 24-week outcome, which corresponds to 42 patients recruited to each of the four treatment arms (control, 20 mg, 40 mg, 80 mg) at each of the two stages. At the end of the first stage, a *t* test statistic for each of the active doses versus control was calculated. If any test statistic was above 2.782, the trial would be stopped for efficacy and that dose recommended for a phase III trial; if any test statistic was below 0, it would be dropped for futility. If no experimental arm stopped for efficacy and at least one did not stop for futility, then the second stage would recruit 42 additional patients per remaining arm. Final test statistics, using data from both stages, would be compared to a critical value of 2.086.

**Fig. 3:**
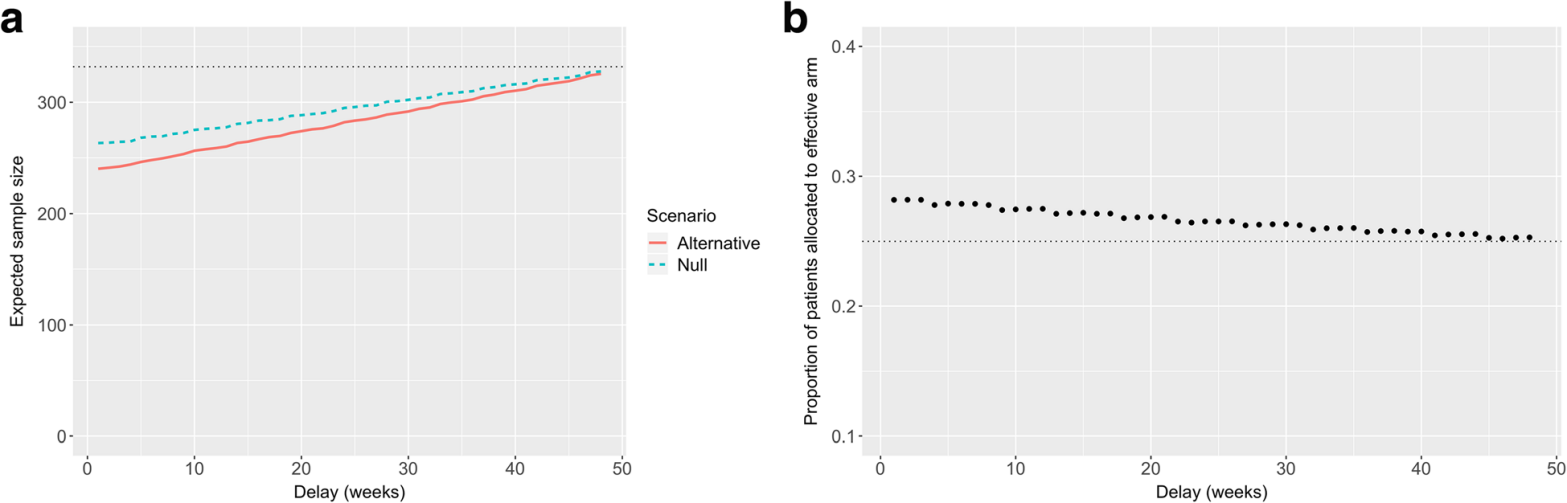
Properties of the TAILoR trial assuming different delay lengths in the endpoint. The actual endpoint was assessed 24 weeks after randomisation. Plotted trial properties were simulated using 10,000 simulation replicates for each potential endpoint delay length between 1 and 48 weeks. **a** Expected sample size averaged over 10,000 simulation replicates for each delay length. Blue dashed line represents the properties of the trial under the null scenario, when all experimental doses have the same efficacy as control; red solid line represents the properties under the alternative scenario, when one dose has a standardised effect of 0.545 and the others have the same efficacy as control. **b** Proportion of patients who were allocated to the effective dose in the alternative scenario (as in a, one dose had a standardised effect of 0.545 and the others 0)

Having delay does hurt the efficiency gain – whilst the required number of first-stage patients were reaching 24 weeks follow-up, second-stage patients were being recruited. We explored the effect of the length of the endpoint delay in simulations. We consider the endpoint delay varying from 1 to 48 weeks and simulated 10,000 trial replicates for each. This allowed us to explore the effect of delay on the statistical properties. We considered two scenarios, namely when all doses had the same effect as control (null scenario) and when two doses had no effect and one dose had a standardized effect of 0.545 (alternative scenario). Figure 3 shows the expected sample size of the trial under each scenario and the proportion of participants allocated to the effective treatment in the alternative scenario. Clearly, the delay had a substantial effect on the expected sample size and the advantages to patients. Nevertheless, with the actual 24-week delay observed in the trial, there was a substantial benefit from the adaptive approach, wherein the sample size needed (on average) was reduced and there was an increase in the proportion of patients allocated to the best treatment.

It should be noted that the time taken to perform the interim analysis (see later section on logistical complexity) was not included in the 24-week delay, and thus, in practice, the delay might have been longer. We consider in a later section logistical issues that may either prolong the interim analysis, require substantially more trial resources, or lower the quality of the information on the endpoint assessed at the interim. Any of these issues will reduce the benefits given by the adaptive design.

## Limitations due to early stopping

Many adaptive designs allow early stopping of the trial or of individual treatment arms, within a MAMS study. This stopping could be for futility, when there is little prospect of a positive finding given the data seen up to that point, or for efficacy when there is already sufficient evidence to conclude the treatment is efficacious.

Generally, as argued above, this early stopping is advantageous. It means that fewer participants are required, on average, the trial can finish quicker, and fewer participants may be allocated to ineffective treatment arms. However, it can also cause a number of problems that may outweigh the advantages. Stopping early for efficacy means that the trial may not provide convincing information on secondary outcomes, safety or subgroup effects. Additionally, there is often scepticism that early stopping might reflect a random high and the treatment effectiveness might not be as great as suggested [18]. Moreover, if the precision of the estimated treatment effects is broad due a reduced sample size, this may be less convincing in terms of changing clinical practice (or National Institute of Health and Care Excellence guidance). Stopping early for futility may also mean that advantages provided by the treatment in a participant subgroup or on an important secondary outcome may be missed; thus, it is important to consider whether stopping early runs the risk of missing out on important information. It is also important to consider what happens to participants allocated to an arm that is stopped early. Should they stop treatment or switch to another arm? For accurate reporting of the dropped arm, they would still be followed, but this may be contrary to arguments in favour of the adaptive approach that appeal to improved patient benefit of trial participants.

## Limitations due to additional administrative and logistical complexity

Another issue that, in our experience, is frequently underestimated by investigators is the additional complexity that an adaptive design causes to the conduct of a trial. To provide an advantage over a non-adaptive design, interim analyses must be conducted quickly and to a high standard. This involves having an effective infrastructure within the trial team that may require considerable investment of resources. For example, return of data for the analysis has to be prompt and complete, and data queries and cleaning have to be to a high standard throughout the trial so that the data snapshot for the interim analysis does not exclude large amounts of pending or incomplete data. In addition, effective communication within the trials team, as well as between trial investigators and the data monitoring committee, is required to understand the impact of the adaptive features.

Any delays in conducting interim analyses or implementing adaptations will reduce the efficiency advantage of an adaptive approach in exactly the same way as using an outcome that takes longer to observe (Fig. 1). To our knowledge, there is no paper systematically investigating how quickly and to what level of quality interim analyses have been performed in adaptive designs.

An example of a trial which has made available information on the time taken is the STAMPEDE trial, a MAMS trial allowing early stopping for lack-of-benefit. In Sydes et al. [19], details are presented about an interim analysis where recruitment to an arm was terminated due to lack of benefit. The time lapse from the database being frozen to the decision to drop the arm being ratified was just over 2 months. This process is impressively quick given that it involves an analysis being conducted as well as meetings of the data monitoring committee and trial steering committee. The decision of the trial steering committee was implemented in sites on the same day. It is likely that the typical length of time taken in adaptive designs is considerably longer.

An additional example of overcoming logistical difficulties is the BATTLE-2 trial, testing four treatments for lung cancer using a Bayesian adaptive randomisation design. In section 4 of Gu et al. [20], the substantial infrastructure for ensuring high-quality information is described.It would be interesting to contrast the above requirements for adaptive designs to the needs for data monitoring in non-adaptive designs. It is our opinion that adaptive trials likely require more resources, but this needs further research.

## Weighing the pros and cons of adaptive designs

Adaptive designs undoubtedly have benefits for improving the efficiency of testing experimental treatments in many situations. However, in other situations, the benefits may be marginal and not sufficient to justify the drawbacks. We believe that it is vital to properly assess the benefits of an adaptive design prior to embarking on one and that no single method (i.e. adaptive or non-adaptive) should be the default for a particular clinical setting. It is important that theoretical work that proposes and promotes adaptive designs clearly lays out any reduction in their reported efficiency benefits when there is substantial delay in outcome evaluation. It is also important to carefully consider reducing the complexity of an adaptive design when the efficiency gains are marginal. For example, in our experience, it is rare that having more than two interim analyses during a trial provides enough additional benefit to justify the additional burden unless the trial is over a very long period of time or involves adding in new arms as the trial progresses.

Having an efficient infrastructure that reliably delivers the promised increase efficiency of adaptive designs will likely increase the financial costs of the trial. This is an area where more research is needed to assess the additional cost of an adaptive design and when is it worthwhile. Once there is more information on costs, and better information on the efficiency provided by adaptive trials in real-world scenarios, investigators and funders should carefully consider which design is most appropriate for the specific setting of the trial. Careful consideration of outcomes, recruitment, data quality and trial complexity should be built into developing the trial design and assessing its properties. Further methodological research is needed to provide specific guidelines about when being adaptive is worthwhile.

## Conclusions

We do not aim to suggest that adaptive designs should not be used. In fact, they are frequently the best option for efficiency reasons and from the perspective of trial participants. However, we also wish to ensure it is well understood by investigators that they are not a universal panacea that should be used in all trials. Investigators should carefully consider the potential benefits and drawbacks of the design used as well as whether it is actually feasible to perform with the resources available.

## Data Availability

Data sharing is not applicable to this article as no datasets were generated or analysed during the current study.

## Abbreviations

ISRCTN: International Standard Randomised Controlled Trial Number
MAMS: Multi-arm multi-stage
